# Meniscal degeneration among knees without radiographic osteoarthritis correlates with changes in disease activity and subsequent cumulative damage: Data from the Osteoarthritis Initiative

**DOI:** 10.1016/j.ostima.2025.100264

**Published:** 2025-03-23

**Authors:** Joshua T. Harvey, Timothy E. McAlindon, Jonggyu Baek, Jamie MacKay, Ming Zhang, Grace H. Lo, Shao-Hsien Liu, Charles B. Eaton, Matthew S. Harkey, Julieann C. Patarini, Jeffrey B. Driban

**Affiliations:** a Department of Population and Quantitative Sciences, UMass Chan Medical School, 55 Lake Avenue North, Worcester, MA 01655, USA; b Division of Rheumatology, Department of Medicine, UMass Chan Medical School, 55 Lake Avenue North, Worcester, MA 01655, USA; c Department of Radiology, University of Cambridge, Box 218, Cambridge Biomedical Campus, Cambridge CB2 0QQ, UK; d Norwich Medical School, University of East Anglia, Norwich Research Park, Norwich, NR4 7TJ, UK; e Department of Computer Science, Boston University, 111 Cummington Mall, Boston, MA 02215, USA; f Department of Medicine, Baylor College of Medicine, One Baylor Plaza, Houston, TX 77030, USA; g Medical Care Line and Research Care Line, Houston VA HSR&D Center for Innovations in Quality, Effectiveness and Safety, Michael E. DeBakey Medical Center, 2002 Holcombe Blvd, Houston, TX 77030, USA; h Center for Primary Care and Prevention, Kent Hospital, 111 Brewster St, Pawtucket, RI 02860, USA; i Brown University School of Public Health, 121 South Main Street, Providence, RI 02903, USA; j Department of Kinesiology, Michigan State University, 308 W. Circle Drive, East Lansing, MI 48824, USA

**Keywords:** MRI, Meniscus, Articular cartilage, Bone, Effusion, Synovitis

## Abstract

**Objective:**

To explore the relationship between meniscal degeneration (intrameniscal signal alteration without a tear) and future osteoarthritis pathology as measured by composite scores based on magnetic resonance imaging (MRI) of disease activity (bone marrow lesions and effusion-synovitis volumes) and cumulative damage (articular cartilage damage).

**Design:**

This analysis involved 225 participants from the Osteoarthritis Initiative with intact menisci (defined as normal or meniscal degeneration without tear) on MRI and no radiographic knee osteoarthritis at baseline. We used longitudinal MRIs from an existing study to calculate disease activity and cumulative damage. We used robust regression models to assess the association between baseline meniscal degeneration (exposure) and disease activity or cumulative damage at baseline and four annual follow-up visits (outcomes), adjusting for sex, race, age, static alignment, and body mass index.

**Results:**

Our sample included 110 participants with normal menisci (77 % women, average age 55 [SD 7]) and 115 with meniscal degeneration (60 % women, average age 61 [SD 9]). Knees with meniscal degeneration were more likely to have, on average, 0.21 greater disease activity at 12 months than knees with normal menisci (parameter estimate = 0.21, 95 % confidence interval (CI) = 0.09, 0.33). The association persisted over time. In contrast, the association between meniscal degeneration and cumulative damage only became statistically significant at the 48-month visit (parameter estimate = 0.74, 95 % CI = 0.18, 1.31).

**Conclusions:**

Meniscal degeneration was related to worsening disease activity earlier than articular cartilage damage among knees without radiographic osteoarthritis. Meniscal degeneration and disease activity are promising biomarkers for early detection and monitoring of osteoarthritis, and may inform potential early intervention strategies.

## Introduction

The menisci of the knee are important in joint health because they distribute force load and absorb shock [1]. Meniscal pathology is a risk factor for knee osteoarthritis incidence and progression [1]. Specifically, meniscal degeneration – intrameniscal signal alterations without a discrete tear – may predispose knees without radiographic osteoarthritis to develop a meniscal tear and an accelerated onset and progression of osteoarthritis [2,3]. The natural history of osteoarthritis-related biomarkers among knees with meniscal degeneration remains poorly understood.

Understanding the relationship between meniscal degeneration and osteoarthritis-related biomarkers in knees without radiographic osteoarthritis is essential for improving early detection, monitoring of disease progression, and developing intervention strategies to prevent or slow the progression of this debilitating condition. Disease activity, a dynamic composite biomarker representing bone marrow lesions (BMLs) and effusion-synovitis, and cumulative damage, which represents the gradual progression of articular cartilage damage [4], are two promising composite biomarkers that we have previously developed and validated [4,5] to help conceptualize osteoarthritis onset and progression.

The objective of this study was to examine the relationship between meniscal degeneration in knees with no signs of radiographic osteoarthritis and the development of osteoarthritis pathology as measured by disease activity and cumulative damage. In these exploratory analyses, we sought preliminary evidence for a theoretical framework for understanding the early stages of osteoarthritis, in which meniscal degeneration is a common and very early indication of disease, preceding increased disease activity and worsening cumulative damage.

## Materials and methods

### Osteoarthritis Initiative (OAI)

The OAI enrolled 4796 individuals who had or were at risk for symptomatic knee osteoarthritis across four clinical sites. The recruitment occurred from February 2004 to May 2006 at four clinical sites: Ohio State University, University of Maryland and John Hopkins, Memorial Hospital of Rhode Island, and University of Pittsburgh. The OAI enrolling sites and the OAI coordinating center at the University of California, San Francisco received institutional review board approval prior to the study. All participants provided informed consent before OAI participation. Using the OAI data, we conducted an exploratory data analysis of data from a nested case-control study [6].

### Original case-control sample

In our initial study, we examined participants without radiographic knee osteoarthritis at the OAI baseline and grouped them based on the rate of radiographic onset and progression during the first four years of the OAI. Central OAI readers graded annually-collected weight-bearing, fixed-flexion posterior-anterior knee radiographs to monitor radiographic disease progression (files: kXR_SQ_BU##_SAS [versions 0.6, 1.6, 3.5, 5.5, and 6.3]; intrarater agreement: weighted kappa=0.70 to 0.80). At baseline, all participants had at least one knee without radiographic osteoarthritis (i.e., Kellgren-Lawrence (KL) grade 0 or 1). We then separated the OAI participants into three groups based on disease progression: 1) *accelerated knee osteoarthritis*: progressed to KL grade 3 or 4 within 4 years (*n* = 125); 2) *typical knee osteoarthritis*: any other KL grade increase within 4 years (*n* = 187); 3) *no knee osteoarthritis*: KL grade remained the same over 4 years (*n* = 1325) [[6], [7], [8]]. Participants with accelerated knee osteoarthritis included anyone who met the criteria, regardless of the baseline radiographic severity in the contralateral knee. Individuals with typical or no knee osteoarthritis had no radiographic knee osteoarthritis in either knee at baseline. Participants were matched by sex in the typical and no osteoarthritis groups to participants in the accelerated osteoarthritis group (i.e., 125 people per group; 375 total) [[6], [7], [8]].

All 375 participants contributed data from one knee. For those with accelerated or typical osteoarthritis, the first knee to be affected was selected. For participants without osteoarthritis, we selected the same knee as the matched member from the accelerated knee osteoarthritis group [[6], [7], [8]].

### Eligible sample for exploratory analyses

This exploratory analysis was based on data from the 375 participants, as described above. From there, we set forth additional inclusion and exclusion criteria. We included only participants with intact menisci defined as having “normal” menisci or “meniscal degeneration” on MR images, and without meniscal extrusion at the OAI baseline visit (*n* = 225).

### Magnetic resonance imaging acquisition

The MR images were collected at the OAI sites using Siemens 3.0 Tesla Trio MR systems and knee coils. Study-certified, licensed MR technicians regularly conducted quality control measurements to maintain quality standards and consistency in the MR equipment. Acquisitions included a sagittal intermediate-weighted fat-suppressed sequence (field of view=160 mm, slice thickness=3 mm, skip=0 mm, flip angle=180 °, echo time=30 ms, recovery time=3200 ms, 313×448 matrix, x-resolution=0.357 mm, y-resolution=0.511 mm), which was used to measure BML and effusion-synovitis volumes. Cartilage damage was quantified using a 3-dimensional dual-echo steady-state (3D DESS) sequence: field of view=140 mm, slice thickness=0.7 mm, skip=0 mm, flip angle=25 °, echo time=4.7 ms, recovery time=16.3 ms, 307×384 matrix, x-resolution=0.365 mm, y-resolution=0 .456mm.

### Semi-quantitative meniscal status

Two radiologists used a modified classification system from the International Society of Arthroscopy, Knee Surgery, and Orthopedic Sports Medicine [6] to evaluate meniscal status at the OAI baseline. Radiologist 1 evaluated 255 knees, and radiologist 2 assessed 120 cases. They looked at the body and anterior/posterior horns of the medial and lateral menisci, categorizing their findings into ten types: normal, meniscal degeneration, radial tear, maceration, complex tear, horizontal tear, flap horizontal tear, vertical longitudinal tear, morphologic deformity, and vertical flap tear [9]. They noted any root tears as well. The current analyses focused on participants with intact menisci at baseline, meaning either normal or showing some degeneration (intrameniscal signal alterations). In a reliability set, the two readers agreed on 83 % of the observations for normal or meniscal degeneration in the six meniscal regions among 24 knees (kappa = 0.54, 95 % confidence interval = 0.37 to 0.71). To calculate the kappa statistic, we pooled the six regions because several regions had multiple cells with small sample sizes (<2). Furthermore, within each region, there was a significant imbalance because of a high prevalence of agreement on “normal” by both readers.

### Measuring bone marrow lesions

One reader used a validated semi-automated software to measure BML volumes using an intermediate-weighted fat-suppressed MR sequence in four regions: the medial and lateral tibia and femur [9,10]. The reader manually identified a crude boundary of each bone, and the program automatically identified the precise bone borders. The program then performed a thresholding and curve evolution process twice to segment regions of high-signal intensity. The program eliminated false-positive areas of high-signal intensity by requiring a BML to meet two criteria: 1) the distance between the BML segmented boundary and the articular surface should be ≤ 1 cm [9,11]; and 2) BMLs must appear on at least two images. An experienced reader reviewed all measurements. The reader had excellent intra-reader reliability (intra-class correlation coefficients [ICC; 3,1 model] = 0.91). The primary BML outcomes were four regional BML volumes that represented the sum of the BML volume within each anatomic region (medial and lateral distal femur and proximal tibia).

### Measuring effusion-synovitis

One reader measured effusion-synovitis using a validated semi-automated software on the intermediate-weighted fat-suppressed sequence [7]. They used the software to manually adjust thresholds to segment regions of high-signal intensity and remove irrelevant areas of high-signal intensity (e.g., blood vessels, BMLs). The reader had excellent intra-reader reliability (ICC [3,1] =0.96). Our primary effusion-synovitis volume was one volume that represented effusion-synovitis throughout the entire knee.

### Calculating disease activity score

We calculated disease activity based on a validated algorithm [4,5]. First, we adjusted each MR-based measure for an individual's bone width (distance between medial and lateral femoral epicondyles on the 3D DESS MR sequence). This adjustment allowed us to account for variations in knee size. Next, we standardized each measure by subtracting the mean from an independent reference sample and dividing it by the standard deviation from that sample [4,5]. This step ensures that all measurements are on the same scale to enable calculation of disease activity and enhance interpretability. The reference sample included 197 knees from the OAI: 93 % had moderate-severe radiographic knee osteoarthritis (KL grade = 3 or 4), and the average WOMAC knee pain score was 5.0 (SD=3.6; Supplemental Table 1). We used this reference sample to ensure that the disease activity scale can be interpreted consistently across research projects. Finally, we summed the standardized measures in effusion-synovitis and the BML volumes for the four regions (medial and lateral; distal femur and proximal tibia). A disease activity score of 0 approximated the average score for the reference sample; lower scores (negative scores) indicated milder disease, greater values indicated worse disease.

### Quantifying cartilage damage index

A single reader used a custom semi-automated program to quantify the tibiofemoral cartilage damage index (CDI), a valid measure of cartilage thickness [12,13].The reader measured cartilage thickness at 36 informative locations in the medial/lateral femur/tibia (i.e., 9 locations in each region) that were automatically located by the semi-automated program based on a standardized coordinate system. These 36 locations were considered informative because they are where cartilage defects are most likely to develop in knee osteoarthritis [12,13]. The semi-automated program produced a CDI value for all four tibiofemoral regions (medial/lateral femur/tibia). The study's principal investigator reviewed all CDI measurements, and the reader had excellent intra-reader reliability (ICC [3,1 model] = 0.86 to 0.99).

### Calculating cumulative damage score

We calculated cumulative damage using our validated algorithm[4,5]. First, we adjusted each MR-based measure for each person's bone width (distance between medial and lateral femoral epicondyles on the 3D DESS MR sequence). This adjustment allowed us to account for variations in knee size. Next, we standardized each measure by subtracting the mean from an independent reference sample (described above) and dividing it by the standard deviation from that sample[4,5]. This step ensures that all measurements are on the same scale to enable the calculation of cumulative damage and to enhance interpretability. We then summed the standardized cartilage measures for the four regions (medial and lateral: distal femur and proximal tibia). Finally, we multiplied by −1 to ensure that higher values indicate more severe disease.

### Clinical data

Baseline demographics were acquired at the OAI baseline visit. These variables included body mass index, age, Western Ontario and McMaster Universities Osteoarthritis Index (WOMAC) pain score, sex (“What is your sex, male or female?”), race (“What is your racial background?”; open-ended question), and static alignment (sex-adjusted femorotibial angle measured on weight-bearing, fixed-flexion posteroanterior knee radiographs. This data is available to the public (File: enrollees, version 23; allclinical00, version 0.2.2; and kXR_FTA_Duryea00, version 0.2).

### Statistical analysis

We generated descriptive statistics for all study variables, including means and standard deviations for continuous variables such as age and frequencies and percentages for categorical variables such as race. Based on the sample size and the right-skewed distribution of disease activity, we used robust regression models with M estimation and Huber weights to explore the association between baseline meniscal status (binary: normal or degenerative) and disease activity or cumulative damage at baseline and at each of the first four annual follow-up visits as well as the 48-month change in each score. All models were adjusted for sex (male/female), race (White/Black or African American/Other), baseline age (continuous), baseline static alignment (femorotibial angle; continuous), and baseline body mass index (continuous). We fitted separate models for each visit. In the tables, we report the adjusted means (95 % CI) and the parameter estimate (95 % CI). The parameter estimate for each model describes the adjusted average difference in cumulative damage or disease activity between subjects with meniscal degeneration versus those without degeneration. Another way to conceptualize the parameter estimate is the mean “total difference across all 7 standardized components” between those with or without meniscal degeneration. We conducted sensitivity analyses to assess the robustness of our results by switching from Huber weights to bisquare weights. To better understand whether certain components of the composite scores influence these results, we performed similar analyses with the two main components of disease activity (total knee BML volume and effusion-synovitis volume)as the outcomes and the four main components of cumulative damage (cartilage damage index in the medial femur, medial tibia, lateral femur, and lateral tibia). Lastly, we explored the association between baseline meniscal damage and disease activity or cumulative damage among people without contralateral radiographic knee osteoarthritis. All analyses were performed using SAS Enterprise 8.3 (Cary, NC, USA).

## Results

Our sample included 110 participants with normal menisci (77 % female, average age 55 ± 7 years) and 115 with meniscal degeneration (60 % female, average age 61 ± 9 years; Table 1). At baseline, the disease activity score ranged from −2.91 to 6.07 (5th percentile = −2.75 and 95th percentile = −0.83), and the cumulative damage score ranged from −10.33 to 5.61 (5th percentile = −5.88 and 95th percentile = 3.73). At the 48-month OAI visit, the disease activity score ranged from −2.88 to 7.38 (5th percentile = −2.63 and 95th percentile = 0.65), and the cumulative damage score ranged from −9.59 to 6.63 (5th percentile = −5.15 and 95th percentile = 4.45).

**Table 1:** Baseline characteristics of people with and without meniscal degeneration at baseline.

	**Normal Menisci** **(*n*=110)**	**Menisci with Degeneration** **(*n*=115)**
	**n ( %) or Mean (SD)**	**n (%) or Mean (SD)**
Females	85 (77 %)	69 (60 %)
White	92 (84 %)	101 (88 %)
Black or African American	16 (15 %)	11 (10 %)
Age (years)	55.1 (7.1)	61.0 (8.6)
Static alignment (femoro-tibial angle, degrees)*	−0.8 (1.8)	−0.7 (2.0)
Body mass index (kg/m^2^)	27.7 (4.8)	28.7 (4.8)

⁎ Femoro-tibial angle: Varus malalignment showed more negative values than knees with valgus malalignment.

Table 2 shows the association between baseline meniscal degeneration of both disease activity and cumulative damage at baseline and future visits. Knees with meniscal degeneration were significantly more likely to have greater disease activity at 12 months (parameter estimate=0.21, 95 % CI=0.09 to 0.33). Hence, on average, disease activity was 0.21 greater among knees with meniscal degeneration than those without. Alternatively, this could be interpreted to mean that the estimated total difference across all 7 standardized components was 0.21 between knees with or without meniscal degeneration. The association between meniscal degeneration and disease activity persisted over the next three years (parameter estimates=0.28 to 0.41).

**Table 2:** Baseline meniscal signal alterations relate to disease activity and cumulative damage cross-sectionally and at future visits.

	Disease Activity^1^			Cumulative Damage^1^		
	Normal Menisci (*n* = 110)	Menisci with Degeneration (*n* = 115)	Parameter Estimate^2^ (95 % CI)	Normal Menisci (*n* = 110)	Menisci with Degeneration (*n* = 115)	Parameter Estimate^2^ (95 % CI)
Visit	Mean^2^ (95 % CI)	Mean^2^ (95 % CI)		Mean^2^ (95 % CI)	Mean^2^ (95 % CI)	
Baseline (*n* = 225)	−2.30 (−2.38, −2.23)	−2.22 (−2.29, −2.16)	0.08 (−0.02, 0.18)	−0.18 (−0.58, 0.22)	0.23 (−0.11, 0.57)	0.41 (−0.11, 0.93)
12 month (*n* = 221)	−2.26 (−2.35, −2.16)	−2.04 (−2.12, −1.97)	0.21 (0.09, 0.33)	0.5 (−0.37, 0.46)	0.46 (0.11, 0.80)	0.41 (−0.13, 0.95)
24 month (*n* = 221)	−2.10 (−2.22, −1.99)	−1.82 (−192, −1.73)	0.28 (0.13, 0.43)	0.20 (−0.21, 0.60)	0.67 (0.32, 1.01)	0.47 (−0.06, 1.01)
36 month (*n* = 205)	−2.03 (−2.17, −1.89)	−1.62 (−1.75, −1.50)	0.41 (0.22, 0.60)	0.35 (−0.07, 0.78)	0.86 (0.49, 1.23)	0.51 (−0.06, 1.07)
48 month (*n* = 220)	−2.02 (−2.16, −1.89)	−1.71 (−1.83, −1.59)	0.31 (0.13, 0.49)	0.37 (−0.61, 0.80)	1.11 (0.74, 1.48)	0.74 (0.18, 1.31)
Change 0 to 48 months	0.22 (0.11, 0.34)	0.49 (0.39, 0.59)	0.27 (0.11, 0.42)	0.46 (0.31, 0.61)	0.66 (0.53, 0.78)	0.20 (0.00, 0.40)

1 Higher values indicate worse damage. Negative values represent milder disease activity or cumulative damage than the average of a reference sample, among whom 93 % had moderate-severe radiographic knee osteoarthritis (KL grade = 3 or 4), and the average WOMAC knee pain score was 5.0 (SD=3.6; Supplemental Table 1).

2 Adjusted for sex (2 levels), race (3 levels), baseline age, baseline static alignment (adjusted FTA), and baseline body mass index.

In contrast, no significant association was found between meniscal degeneration and cumulative damage at the 12-, 24-, or 36-month visit, but at the 48-month visit the association had become statistically significant (parameter estimate=0.74, 95 % CI=0.18, 1.31). Baseline meniscal degeneration was associated with 48-month changes in cumulative damage and disease activity (Table 2). The overall findings were consistent with bisquare weights instead of Huber weights.

Within the components of disease activity (Table 3), we found that knees with meniscal degeneration were significantly more likely to have greater effusion-synovitis and bone marrow lesion volumes at 12 months. These associations persisted over the subsequent three years (Table 3). Baseline meniscal degeneration was associated with 48-month effusion-synovitis volume change but not BML volume change (Table 3).

**Table 3:** Baseline meniscal signal alterations relate to effusion-synovitis and bone marrow lesion volumes cross-sectionally and at future visits.

	Effusion-Synovitis Volume (cm^3^)			Bone Marrow Lesion Volume (cm^3^)		
	Normal Menisci (*n* = 110)	Menisci with Degeneration (*n* = 115)	Parameter Estimate^1^ (95 % CI)	Normal Menisci (*n* = 110)	Menisci with Degeneration (*n* = 115)	Parameter Estimate^1^ (95 % CI)
Visit	Mean^1^ (95 % CI)	Mean^1^ (95 % CI)		Mean^1^ (95 % CI)	Mean^1^ (95 % CI)	
Baseline (*n* = 225)	7.11 (6.38, 7.84)	7.68 (7.06, 8.30)	0.57 (−0.39, 1.53)	0.56 (0.46, 0.66)	0.65 (0.56, 0.73)	0.09 (−0.04, 0.22)
12 month (*n* = 221)	7.40 (6.53, 8.27)	9.52 (8.79, 10.26)	2.12 (0.98, 3.26)	0.66 (0.53, 0.79)	0.85 (0.74, 0.97)	0.19 (0.02, 0.37)
24 month (*n* = 221)	8.73 (7.62, 9.83)	11.19 (10.25, 12.13)	2.46 (1.01, 3.92)	0.79 (0.63, 0.95)	1.0 (1.78)	0.22 (0.02, 0.43)
36 month (*n* = 205)	9.39 (8.06, 10.73)	12.88 (11.72, 14.04)	3.49 (1.71, 5.26)	0.87 (0.68, 1.07)	1.26 (1.09, 1.43)	0.39 (0.13, 0.64)
48 month (*n* = 220)	9.88 (8.52, 11.24)	12.91 (11.76, 14.07)	3.04 (1.26, 4.81)	0.71 (0.56, 0.85)	0.95 (0.83, 1.08)	0.25 (0.05, 0.44)
Change 0 to 48 months	2.08 (1.05, 3.11)	4.65 (3.77, 5.52)	2.57 (1.22, 3.92)	0.08 (−0.06, 0.21)	0.19 (0.08, 0.31)	0.12 (−0.06, 0.30)

1 Adjusted for sex (2 levels), race (3 levels), baseline age, baseline static alignment (adjusted FTA), and baseline body mass index.

Within the components of cumulative damage (Supplemental Tables 2 and 3), we found that the results in the medial femur agreed with our primary observation that meniscal degeneration is associated with both greater cartilage damage and worsening changes in cartilage at the 48-month visit (Supplemental Table 2). We observed no statistically significant associations between baseline meniscal damage and cartilage in the medial tibia or lateral femur. Baseline meniscal degeneration consistently corelated with greater cartilage damage in the lateral tibia at each visit but not with changes in lateral tibial cartilage.

When we excluded people with contralateral radiographic knee osteoarthritis, our disease activity analyses agreed with our overall findings (Supplemental Table 4). In contrast, we observed no statistically significant associations with cumulative damage.

## Discussion

Overall, our findings suggest that meniscal degeneration relates to disease activity before it relates to cumulative damage. This supports a theoretical hypothesis that changes in disease activity, especially effusion-synovitis, likely precede changes in cumulative damage in knees with meniscal degeneration. Additionally, these results support the pathogenic role of meniscal degeneration in osteoarthritis. Hence, this represents a paradigm shift in conceptualizing the pathogenesis of osteoarthritis, in which meniscal degeneration leads to inflammatory pathways and then cartilage damage, possibly mediated by an innate immune response and biomechanics.

Meniscal degeneration is a critical finding in knees without radiographic evidence of osteoarthritis, as it is associated with a poor prognosis, including an elevated risk of meniscal tears[2,3] and an accelerated onset and progression of osteoarthritis[3]. Our findings build on this evidence by demonstrating that meniscal degeneration is associated with heightened disease activity, particularly worsening effusion-synovitis, at earlier stages than cumulative structural damage. These findings align with evidence that greater effusion-synovitis and larger BML volumes precede the development of an accelerated type of knee osteoarthritis. Meanwhile, cartilage damage is a lagging indicator that worsens during the radiographic onset of accelerated knee osteoarthritis[6,7]. These findings also complement prior research identifying effusion-synovitis, BMLs, and medial meniscal damage as early pathological features linked to incident radiographic osteoarthritis[[14], [15], [16], [17]]. In contrast, medial compartment cartilage defects were later features that preceded the onset of osteoarthritis. These prior studies often focused on discrete tears or meniscal extrusion as risk factors for osteoarthritis. Hence, the current analyses add further support that meniscal degeneration should be considered a significant pathologic finding in early stages of knee osteoarthritis. Together, these findings highlight the central role of meniscal degeneration in the pathogenesis of osteoarthritis, potentially driving an inflammatory response reflected in early effusion-synovitis and altered joint loading. These processes may contribute to the development of BMLs and eventual cartilage damage, underscoring the importance of targeting disease activity to mitigate the progression of osteoarthritis.

A limitation of our study is that while we hypothesize that disease activity is more sensitive because it represents a more dynamic aspect of the disease process, we cannot rule out that cumulative damage appeared less sensitive because of measurement challenges. Secondly, we used a convenience sample from a prior case-control study to explore our hypothesis, which confirmed the presence of insightful associations but limited our ability to make inferences about the strength of the association in a more generalizable population. The current results offer promising evidence to encourage future studies to continue testing this theoretical framework for early-stage osteoarthritis in studies with 1) longitudinal data before and after the onset of meniscal degeneration, 2) more robust analyses to assess the trajectory over time, 3) a more generalizable study sample, and 4) an external dataset to further validate these findings.

We built on prior research to provide promising evidence that meniscal degeneration is important to the pathogenesis of knee osteoarthritis. Meniscal degeneration may elicit early progression in disease activity, especially effusion-synovitis, followed by BMLs and later cartilage damage. Disease activity may offer a promising biomarker of early-stage osteoarthritis, and meniscal degeneration may be a therapeutic target during early-stage osteoarthritis.

## Authors’ disclosures

**Timothy E. McAlindon** declares he is a consultant for Sanofi, Kolon TissueGene, Medidata, Organogenesis, and is the owner of Ambulomics and Arthometrics. Timothy E. McAlindon holds a patent for Objective Assessment of Joint Damage, US-20220202356, 2020.

**Jeffrey B. Driban** holds a patent for Objective Assessment of Joint Damage, US-20220202356, 2020.

**Ming Zhang** holds a patent for Objective Assessment of Joint Damage, US-20220202356, 2020.

**Grace Lo** declares that this work was supported in part with resources at the VA's Health Services Research and Development Service Center for Innovations in Quality, Effectiveness, and Safety (#CIN 13-413) at the Michael E. DeBakey VA Medical Center, Houston, TX. The views expressed in this article are those of the authors and do not necessarily represent the views of the Department of Veterans Affairs.

**The other authors** declare that they have no conflicts of interest.

These analyses were financially supported by a grant from the National Institute of Arthritis and Musculoskeletal and Skin Diseases of the National Institutes of Health under Award Numbers R01-AR076411 and R01-AR065977.

The OAI is a public-private partnership comprised of five contracts (N01-AR-2–2258; N01-AR-2–2259; N01-AR-2–2260; N01-AR-2–2261; N01-AR-2–2262) funded by the National Institutes of Health, a branch of the Department of Health and Human Services, and conducted by the OAI Study Investigators. Private funding partners include Merck Research Laboratories; Novartis Pharmaceuticals Corporation; GlaxoSmithKline; and Pfizer, Inc. Private sector funding for the OAI is managed by the Foundation for the National Institutes of Health. This manuscript was prepared using an OAI public use data set and does not necessarily reflect the opinions or views of the OAI investigators, the NIH, or the private funding partners. This work was supported in part with resources at the VA's Health Services Research and Development Service Center for Innovations in Quality, Effectiveness, and Safety (#CIN 13-413) at the Michael E. DeBakey VA Medical Center, Houston, TX. The views expressed in this article are those of the authors and do not necessarily represent the views of the Department of Veterans Affairs. The funding sources had no role in study design; in the collection, analysis, and interpretation of data; in the writing of the report; nor in the decision to submit the article for publication. The funding sources had no role in study design; in the collection, analysis, and interpretation of data; in the writing of the report; nor in the decision to submit the article for publication.

## Declaration of competing interest

The authors declare that they have no known competing financial interests or personal relationships that could have appeared to influence the work reported in this paper.

## Appendix Supplementary materials

Image, application 1
