# Supplementary material for: Meniscal degeneration among knees without radiographic osteoarthritis correlates with changes in disease activity and subsequent cumulative damage: Data from the Osteoarthritis Initiative
Source: Osteoarthr Imaging. 2025 Mar 23;5(2):100264. doi: 10.1016/j.ostima.2025.100264 (PMC12439794; doi:10.1016/j.ostima.2025.100264)
Supplement: Supplementary file 1 [file mmc1.docx]

| **Supplemental Table 1. Descriptive characteristics of the reference population used to standardize the MRI measurements (n = 197)** | |
| --- | --- |
|  | Number and(%) or mean (SD) |
| **Baseline Characteristics** |  |
| Age (years) | 61.2 (8.8) |
| Body mass index (kg/m^2^) | 30.1 (5.1) |
| WOMAC pain (0-20) | 5.0 (3.6) |
| KL grade |  |
| 0 | 1 (<1%) |
| 1 | 9 (5%) |
| 2 | 83 (42%) |
| 3 | 101 (51%) |
| 4 | 3 (2%) |
| **Clinical Outcomes** |  |
| KL grade progression during the subsequent 48 months | 56 (29%) |
| WOMAC pain worsening by more than 3 points during the subsequent 48 months | 31 (16%) |
| Note: The reference sample was selected from our original validation cohort (U01 AR067168). We used one visit from the Osteoarthritis Initiative per knee. | |

| **Supplemental Table 2. Baseline meniscal signal alterations relate to cartilage damage index in the medial femur and medial tibia cross-sectionally and at future visits** | | | | | | |
| --- | --- | --- | --- | --- | --- | --- |
|  | Medial Femur | | | Medial Tibia | | |
|  | Normal Menisci  (n = 110) | Menisci with Degeneration  (n = 115) | Parameter Estimate^1^  (95% CI) | Normal Menisci  (n = 110) | Menisci with Degeneration  (n = 115) | Parameter Estimate^1^  (95% CI) |
| Visit | Mean^1^ (95% CI) | Mean^1^ (95% CI) |  | Mean^1^ (95% CI) | Mean^1^ (95% CI) |  |
| Baseline (n = 225) | 1659 (1602, 1715) | 1649 (1601, 1697) | -9 (-83, 65) | 736 (705, 767) | 728 (702, 754) | -8 (-48, 33) |
| 12 month (n = 221) | 1628 (1569, 1688) | 1620 (1570, 1669) | -9 (-86, 69) | 719 (686, 753) | 711 (683, 739) | -8 (-52, 36) |
| 24 month (n = 221) | 1601 (1544, 1658) | 1590 (1541, 1638) | -12 (-87, 64) | 704 (670, 738) | 691 (662, 719) | -13 (-58, 31) |
| 36 month (n = 205) | 1615 (1558, 1672) | 1552 (1502, 1602) | -63 (-139, 13) | 695 (663, 727) | 671 (643, 699) | -25 (-67, 18) |
| 48 month (n = 220) | 1589 (1529, 1648) | 1495 (1445, 1546) | **-93 (-170, -16)** | 690 (657, 722) | 655 (627, 683) | -35 (-77, 8) |
| Change 0 to 48 months | -51 (-89, -13) | -102 (-135, -69) | **-51 (-101, -1)** | -37 (-56, -18) | -58 (-74, -42) | -21 (-46, 4) |
| 1. Adjusted for sex (2 levels), race (3 levels), baseline age, baseline static alignment (adjusted FTA), baseline body mass index  Smaller cartilage damage index values indicate more severe disease. | | | | | | |

| **Supplemental Table 3. Baseline meniscal signal alterations relate to cartilage damage index in the lateral femur and lateral tibia cross-sectionally and at future visits** | | | | | | |
| --- | --- | --- | --- | --- | --- | --- |
|  | Lateral Femur | | | Lateral Tibia | | |
|  | Normal Menisci  (n = 110) | Menisci with Degeneration  (n = 115) | Parameter Estimate^1^  (95% CI) | Normal Menisci  (n = 110) | Menisci with Degeneration  (n = 115) | Parameter Estimate^1^  (95% CI) |
| Visit | Mean^1^ (95% CI) | Mean^1^ (95% CI) |  | Mean^1^ (95% CI) | Mean^1^ (95% CI) |  |
| Baseline (n = 225) | 2112 (2048, 2175) | 2115 (2061, 2169) | 3 (-80, 86) | 934 (895, 972) | 874 (841, 907) | -60 (-110, -9) |
| 12 month (n = 221) | 2102 (2039, 2165) | 2107 (2055, 2160) | 5 (-77, 88) | 914 (876, 952) | 850 (818, 882) | -64 (-114, -14) |
| 24 month (n = 221) | 2103 (2035, 2171) | 2109 (2051, 2167) | 6 (-83, 96) | 905 (866, 944) | 829 (796, 862) | -76 (-127, - 24) |
| 36 month (n = 205) | 2079 (2013, 2144) | 2082 (2025, 2139) | 3 (-84, 90) | 880 (839, 920) | 814 (779, 849) | -66 (-120, -12) |
| 48 month (n = 220) | 2104 (2038, 2170) | 2082 (2026, 2138) | -22 (-108, 65) | 878 (836, 921) | 801 (765, 838) | -77 (-133, -21) |
| Change 0 to 48 months | -8 (-46, 29) | -13 (-45, 19) | -5 (-54, 44) | -45 (-63, -27) | -58 (-73, -43) | -13 (-37, 10) |
| 1. Adjusted for sex (2 levels), race (3 levels), baseline age, baseline static alignment (adjusted FTA), and baseline body mass index  Smaller cartilage damage index values indicate more severe disease. | | | | | | |

| **Supplemental Table 4. Baseline meniscal signal alterations relate to disease activity and cumulative damage cross-sectionally and at future visits in those without contralateral radiographic knee osteoarthritis** | | | | | | | |
| --- | --- | --- | --- | --- | --- | --- | --- |
|  | Disease Activity^1^ | | | Cumulative Damage^1^ | | | |
|  | Normal Menisci  (n = 104) | Menisci with Degeneration  (n = 82) | Parameter Estimate^2^  (95% CI) | | Normal Menisci  (n = 104) | Menisci with Degeneration  (n = 82) | Parameter Estimate^2^  (95% CI) |
| Visit | Mean^2^ (95% CI) | Mean^2^ (95% CI) |  | | Mean^2^ (95% CI) | Mean^2^ (95% CI) |  |
| Baseline (n = 186) | -2.29 (-2.37, -2.21) | -2.20 (-2.28, -2.13) | 0.09 (-0.02, 0.20) | | -0.07 (-0.51, 0.36) | 0.06 (-0.35, 0.48) | 0.14 (-0.45, 0.72) |
| 12 month (n = 185) | -2.25 (-2.35, -2.16) | -2.02 (-2.11, -1.94) | 0.23 (0.10, 0.35) | | 0.13 (-0.31, 0.57) | 0.31 (-0.11, 0.73) | 0.18 (-0.41, 0.77) |
| 24 month (n = 184) | -2.15 (-2.27, -2.04) | -1.89 (-2.00, -1.77) | 0.27 (0.11, 0.42) | | 0.22 (-0.21, 0.65) | 0.43 (0.01, 0.84) | 0.20 (-0.38, 0.79) |
| 36 month (n = 176) | -2.06 (-2.19, -1.93) | -1.79 (-1.91, -1.66) | 0.27 (0.10, 0.45) | | 0.30 (-0.14, 0.73) | 0.64 (0.20, 1.07) | 0.34 (-0.27, 0.94) |
| 48 month (n = 186) | -2.06 (-2.18, -1.94) | -1.84 (-1.96, -1.73) | 0.22 (0.06, 0.38) | | 0.29 (-0.16, 0.74) | 0.76 (0.33, 1.20) | 0.48 (-0.13, 1.09) |
| Change 0 to 48 months | 0.17 (0.07, 0.27) | 0.32 (0.23, 0.42) | 0.15 (0.03, 0.28) | | 0.36 (0.22, 0.50) | 0.44 (0.30, 0.57) | 0.08 (-0.11, 0.26) |
| 1. Higher values indicate worse damage. Negative values represent milder disease activity or cumulative damage than the average of a reference sample, among whom 93% had moderate-severe radiographic knee osteoarthritis (KL grade = 3 or 4), and the average WOMAC knee pain score was 5.0 (SD=3.6; Supplemental Table 1).  2. Adjusted for sex (2 levels), race (3 levels), baseline age, baseline static alignment (adjusted FTA), and baseline body mass index | | | | | | | |
